# Constitution of the Buffalo Medical Library Association

**Published:** 1883-03

**Authors:** 


### CONSTITUTION OF THE BUFFALO MEDICAL LIBRARY ASSOCIATION.

### ARTICLE I.

*Name.*

This Association shall be called the BUFFALO MEDICAL LIBRARY ASSOCIATION of the City of Buffalo.

### ARTICLE II.

*Objects.*

The objects of this Association are to facilitate access to current medical literature; to provide for ready reference a library of medical journals, books and monographs; to provide for social intercourse among members of the medical profession, and thus to contribute to the advancement of knowledge in medicine and the collateral sciences.

### ARTICLE III.

*Members.*

This Association shall consist of active and subscribing members.

### ARTICLE IV.

*Officers.*

The officers of this Association shall be a President, Vice-President, Secretary, Treasurer, Librarian and four Trustees.

### ARTICLE V.

*Board of Directors.*

The President, Vice-President, Secretary, Treasurer, Librarian and Trustees shall form a Board of Directors, with full power to appropriate funds, enact by-laws, and conduct the affairs of the Association.

### ARTICLE VI.

*Amendment of Constitution.*

There shall be no alteration in this Constitution unless the same shall have been proposed to and recommended by the Board of Directors, at a meeting previous to the annual meeting, and afterwards by two-thirds of the members of the Association present at the annual meeting.

